# Biofilm formation, antimicrobial susceptibility and virulence genes of Uropathogenic *Escherichia coli* isolated from clinical isolates in Uganda

**DOI:** 10.1186/s12879-020-05186-1

**Published:** 2020-06-29

**Authors:** Paul Katongole, Fatuma Nalubega, Najjuka Christine Florence, Benon Asiimwe, Irene Andia

**Affiliations:** 1 Department of Medical Microbiology, College of Health Sciences Makerere University, Kampala, Uganda; 2 Department of Medical Biochemistry, College of Health Sciences Makerere University, Kampala, Uganda; 3 Department of Medicine, College of Health Sciences Makerere University, Kampala, Uganda

**Keywords:** Biofilms, Uropathogenic *E. coli*, Virulence genes, Antimicrobial resistance

## Abstract

**Introduction:**

Uropathogenic *E. coli* is the leading cause of Urinary tract infections (UTIs), contributing to 80–90% of all community-acquired and 30–50% of all hospital-acquired UTIs. Biofilm forming Uropathogenic *E. coli* are associated with persistent and chronic inflammation leading to complicated and or recurrent UTIs. Biofilms provide an environment for poor antibiotic penetration and horizontal transfer of virulence genes which favors the development of Multidrug-resistant organisms (MDRO). Understanding biofilm formation and antimicrobial resistance determinants of Uropathogenic *E. coli* strains will provide insight into the development of treatment options for biofilm-associated UTIs. The aim of this study was to determine the biofilm forming capability, presence of virulence genes and antimicrobial susceptibility pattern of Uropathogenic *E. coli* isolates in Uganda.

**Methods:**

This was a cross-sectional study carried in the Clinical Microbiology and Molecular biology laboratories at the Department of Medical Microbiology, Makerere University College of Health Sciences. We randomly selected 200 Uropathogenic *E. coli* clinical isolates among the stored isolates collected between January 2018 and December 2018 that had significant bacteriuria (> 10^5^ CFU). All isolates were subjected to biofilm detection using the Congo Red Agar method and Antimicrobial susceptibility testing was performed using the Kirby disk diffusion method. The isolates were later subjected PCR for the detection of Urovirulence genes namely; *Pap, Fim, Sfa, Afa, Hly and Cnf,* using commercially designed primers.

**Results:**

In this study, 62.5% (125/200) were positive biofilm formers and 78% (156/200) of these were multi-drug resistant (MDR). The isolates were most resistant to Trimethoprim sulphamethoxazole and Amoxicillin (93%) followed by gentamycin (87%) and the least was imipenem (0.5%). *Fim* was the most prevalent Urovirulence gene (53.5%) followed by *Pap* (21%), *Sfa* (13%), *Afa* (8%), *Cnf* (5.5%) and *Hyl* (0%).

**Conclusions:**

We demonstrate a high prevalence of biofilm-forming Uropathogenic *E. coli* strains that are highly associated with the MDR phenotype. We recommend routine surveillance of antimicrobial resistance and biofilm formation to understand the antibiotics suitable in the management of biofilm-associated UTIs.

## Background

Urinary tract infections (UTIs) are one of the leading causes of morbidity affecting 150 million people each year worldwide [1]. *E.coli* is the most predominant pathogen causing over 80–90% of community-acquired and 30–50% of hospital-acquired UTIs [2]. The ability of Uropathogenic *E.coli* (UPEC) to invade, grow, ascend and persist in the uroepithelium is dependent on the ability to form biofilms and utilize different virulence factors [3]. These factors however are also countered by the hosts defenses such as urinary flow, expression of cytokines e.g. IL-8, Uro-epithelial defensin peptides such as Tamm-Horsfall protein (THP) and low molecular weight oligosaccharides. Hence the pathogenesis of UTIs relies on the balance between bacterial and host factors [4]. Biofilms represent an assemblage of microbial cells that is irreversibly associated with a surface and enclosed in a matrix of primarily polysaccharide material [5]. Biofilms provide a survival strategy to the bacteria by positioning them to effectively use the available nutrients and prevent access to antimicrobial agents, antibodies and white blood cells [6]. They have also been found to harbor a large number of antibiotic inactivating enzymes such as beta-lactamases hence creating an island of antimicrobial resistance [7].

UPEC strains encode a number of virulence genes that are associated with severe or recurrent UTIs, among these include; P fimbriae *(pap),* type1-fimbriae (*fim-H*), afimbrial-adhesin1 *(afa1),* S-fimbriae *(sfa),* hemolysin *(hly),* cytotoxic-necrotizing-factor *(cnf1),* aerobactin among others [8]. These help the organism to colonize the host surfaces, avoid and or subvert host defense mechanisms, injure and or invade host cells and tissues and incite a noxious inflammatory response hence leading to clinical disease [9]. Biofilm forming bacteria produce matrix composed of proteins, extracellular DNA and polysaccharides, these provide several benefits to the bacterial communities including; protection against immune cells, adhesion (facilitated by bacterial adhesins) and structure [10].

Several studies have demonstrated antimicrobial resistance among UPEC with increasing trends to the most commonly used antibiotics such as ciprofloxacin, trimethoprim-sulphamethoxazole among others [11, 12]. These antimicrobial resistance patterns tend to differ from one geographical region to another [13]. Timely and appropriate treatment is crucial in the management of UTIs; however, this should be based on evidence from regional antimicrobial susceptibility results, knowledge of the virulence genes and biofilm formation [14, 15]. Understanding the link between biofilm formation, presence of virulence genes and antimicrobial resistance distribution in UPEC strains is key in designing effective strategies and measures for prevention and management of UTIs especially severe, recurrent and complicated UTIs [14]. In this study, our goal was to determine the biofilm forming capability, presence of virulence genes and antimicrobial susceptibility pattern of UPEC clinical isolates in Uganda.

## Methods

### Bacterial strains and detection of Uropathogenic *E. coli* virulence genes

This was a cross-sectional study carried out from January 2019 to April 2019 at the Department of Medical Microbiology, Makerere University College of health sciences. A total of 200 *E. coli* isolates (collected and stored January to December 2018) that had been recovered from urine samples of patients with UTIs at Mulago National referral hospital outpatient. The isolates were from pure culture, identified and confirmed biochemically using standard laboratory SOPs. The DNA extraction was carried out using boiling lysis method as described by *Reischl* et al *2000* [16]*.*

We used commercially designed primers for *Pap, Fim, Sfa, Afa, Hly, and Cnf* genes adapted from a study by *Ruike Zhao* et al *2015* [17]*.* The conditions and oligonucleotide sequences are as shown in Table 1. The amplified PCR products were visualized by 1.5% ethidium bromide staining after gel electrophoresis. The amplification of virulence genes was carried out in a Thermal Cycler (Eppendorf Master Cycler) under the following PCR conditions; denaturation at 94 °C for 2 min, followed by 30 cycles of denaturation at 94 °C for 60 s, annealing at 63 °C for 30 s, and extension at 72 °C for 90 s, with a final extension at 72 °C for 5 min. Strain J96 was used as positive control for *Pap, Fim, Sfa, Hly, and Cnf* sequences and strain K10 was used as positive control for *Afa*. Distilled water is used as negative control.

**Table 1:** Oligonucleotide primers used for amplification of virulence genes among UPEC isolates

**Primer name**	**Oligonucleotide sequence (5′-3′)**	**Sizes (bp)**	**Gene**
*PapC-F*	GACGGCACTGCTGCAGGGTGTGGCG	328	*papC*
*Pap-C-R*	ATATCCTTTCTGCAGGGATGCAATA		
*Fim-H-F*	TGTACTGCTGATGGGCTGGTC	564	*fimH*
*Fim-H-R*	GGGTAGTCCGGCAGAGTAACG		
*Sfa-F*	CTCCGGAGAACTGGGTGCATCTTAC	410	*Sfa*
*Sfa-R*	CGGAGGAGTAATTACAAACCTGGCA		
*Afa-F*	GCTGGGCAGCAAACTGATAACTCTC	750	*Afa*
*Afa-R*	CATCAAGCTGTTTGTTCGTCCGCCG		
*HlyA-F*	AACAAGGATAAGCACTGTTCTGGCT	1177	*hlyA*
*HlyA-R*	ACCATATAAGCGGTCATTCCCGTCA		
*Cnf1-F*	AAGATGGAGTTTCCTATGCAGGAG	498	*cnf1*
*Cnf1-R*	TGGAGTTTCCTATGCAGGAG		

Primers and sequences adapted from *Ruike Zhao* et al *2015*

### Detection of biofilm formation and antimicrobial susceptibility testing

For all *E. coli* isolates, biofilm formation was detected by Congo red agar method (CRA) as described by *Freeman* et al *1989* [18]. CRA medium was prepared by mixing brain heart infusion broth (Oxoid, UK) 37 g/L, sucrose 50 g/L, agar No. 1 (Oxoid, UK) 10 g/L and Congo red indicator (Oxoid, UK) 8 g/L. The Congo red stain was prepared as a concentrated aqueous solution and autoclaved (121 °C for 15 min) separately from the other medium constituents [18]. Congo red stain was later added to the autoclaved brain heart infusion agar with sucrose at 55 °C. CRA plates were later inoculated with test organisms and incubated at 37 °C for 24 h aerobically. Black colonies with a dry crystalline consistency indicated biofilm production whereas no-biofilm formation was identified as red or pink crystalline colonies. *E. coli* ATCC 25922 was used as positive control and *Staphylococcus aureus* ATCC 25932 as negative control for the CRA method.

Antimicrobial Susceptibility testing was performed using Kirby Bauer Disk diffusion method on Mueller Hinton agar according to the Clinical Laboratory Standard Institute (CLSI) 2014. Antibiotics tested for included; ampicillin10μg, cefuroxime 30μg, amoxicillin-clavulanic acid 20/10μg, gentamycin10μg, trimethoprim-sulphamethoxazole 1.25/23.5μg, chloramphenicol 5μg, ciprofloxacin 5μg, ceftriaxone 30μg, ceftazidime 30μg, meropenem 10ug, nalidixic acid 30μg, and nitrofurantoin 300μg. *E. coli ATCC 25922* was used as positive control for the antimicrobial susceptibility testing.

### Statistical analysis

The data was cleaned, double-checked and exported to STATA version 14 for statistical analysis. The Chi-square test was used to evaluate the correlations between variables*. P- values* of correlations less than 0.05 were considered statistically significant.

### Ethical considerations

The study obtained approval from the Research and Ethics committee of the School of Biomedical Sciences, College of Health Sciences Makerere University (SBS-HDREC-515) and the Uganda National Council of Science and Technology**.**

## Results

**Fig. 1:**
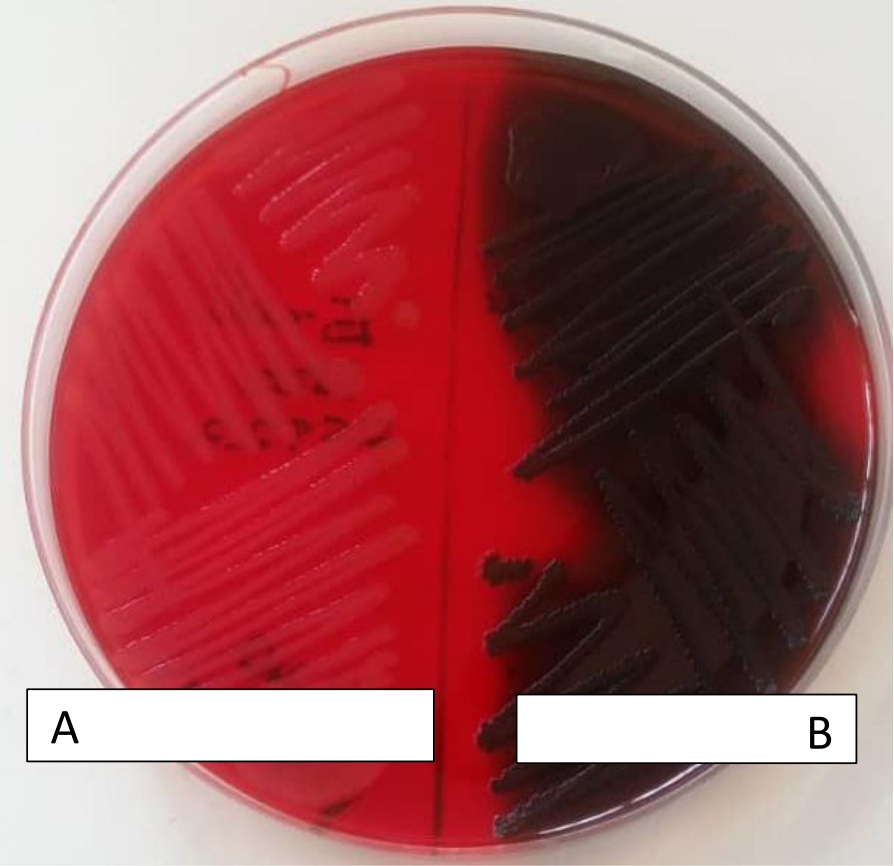
Congo Red Agar plates showing biofilm and none-biofilm forming UPEC isolates

Out of 200 *E. coli* clinical isolates, 125 (62.5%) were able to produce biofilm. All isolates that produced dark crystalline colonies on CRA were considered for positive biofilm formation while those that produced red or pink colonies were considered no biofilm formation as shown in Fig. 1.

**Fig. 2:**
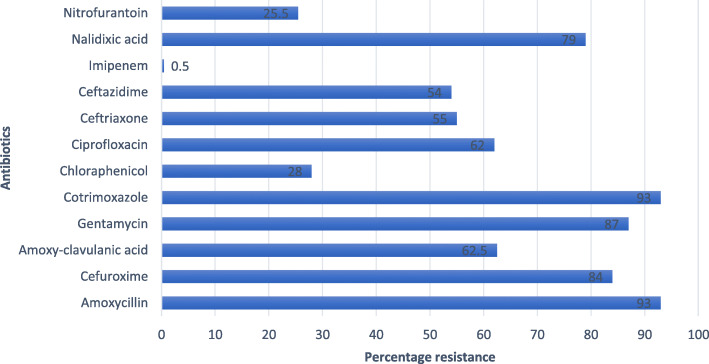
Percentage resistance of Uropathogenic *E.coli* Isolates (*N* = 200) to different antibiotics

Among all the *E. coli* isolates antimicrobial susceptibility pattern varied with resistance with amoxicillin and co-trimoxazole being the highest (93%) followed by, gentamycin (87%), cefuroxime (84%), Nalidixic acid (79%), Amoxicillin clavulanic acid (62.5%), Ciprofloxacin (62%), ceftriaxone (55%),Ceftazidime (54%), chloramphenicol (28%), Nitrofurantoin (25.5%) and Imipenem (0.5%) Fig. 2.

Out of the 200 *E. coli* isolates, 156 (78%) were MDR (multi-drug resistant) i.e. resistance to more than two different antibiotic classes. Biofilm forming *E. coli* isolates were more resistant than the non-biofilm formers with 64% being MDR as compared to 36% among the non-biofilm forming *E. coli* as shown in Table 2 below. The likelihood of biofilm positive UPEC isolates to be MDR was statistically significant with a *p-value* of < 0.05 (within a confidence interval of 95%).

**Table 2:** Multi-drug resistance among biofilm forming and non-biofilm forming UPEC isolates

Biofilm status	MDR phenotype, n (%)
Biofilm formation	100 (64%)
No -Biofilm formation	56 (36%)

**Fig. 3:**
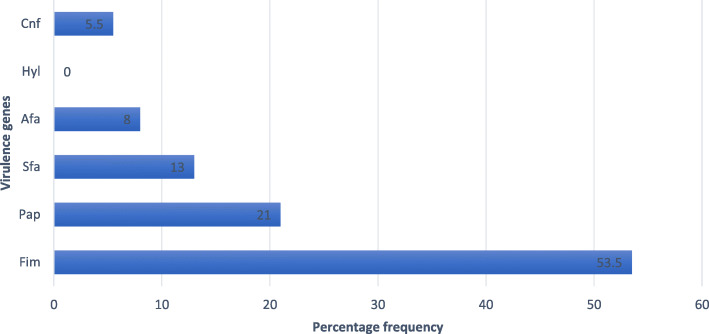
Percentage frequency of Uropathogenic *E.coli* virulence genes

We carried out PCR detection of virulence genes and in this study the prevalence of *fimH, Pap, Sfa, Afa, Hly and Cnf* genes in Uropathogenic *E. coli* was; 53.5, 21, 13, 8%, 0 and 5.5% respectively (Fig. 3).

**Fig. 4:**
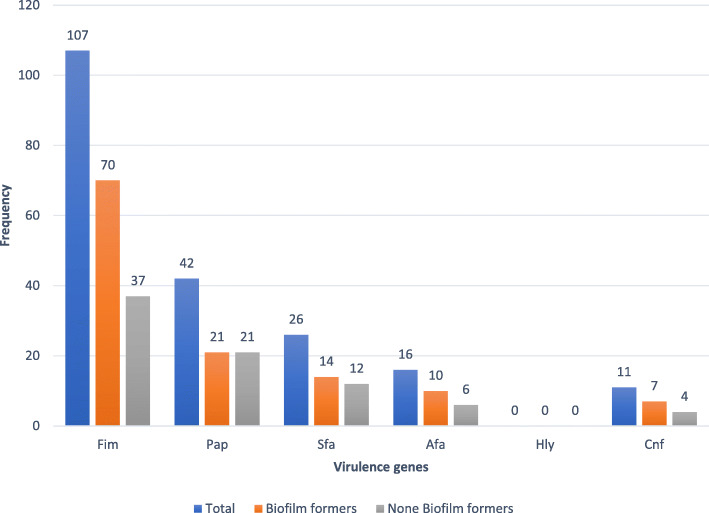
Frequency of virulence genes among Biofilm and None Biofilm forming Uropathogenic *E.coli* isolates

Biofilm formers had more adhesin genes (*Fim, Pap, Sfa and Afa)* than the non-biofilm formers with Fim being the most predominant virulence gene as shown in Fig. 4.

Biofilm production was not significantly associated with the expression of any of the virulence genes.

## Discussion

Biofilm forming bacteria are a common cause of recurrent, and complicated UTIs which are normally associated with MDR bacteria [1]. Understanding the pathogenesis and factors associated with biofilm formation is key to the development of new therapies [19]. In this study we sought to determine the biofilm forming capability, presence of virulence genes and antimicrobial susceptibility pattern of UPEC clinical isolates in Uganda.

Among 200 *E. coli* isolates subjected to biofilm production, the majority of the isolates,125 (62.5%) were biofilm formers on Congo Red Agar (CRA). This was similar to many previous studies [20–22]. In this study we carried out antibiotic susceptibility pattern on all UPEC isolates in their planktonic state. The biofilm-forming isolates showed maximum resistance to Ampicillin and Cotrimoxazole (93%) followed by Gentamycin (87%), Cefuroxime (84%) and Nalidixic Acid (79%). Though resistance to different antibiotics was generally high, biofilm forming organisms were more MDR (64%) compared to non-biofilm formers and this was statistically significant with a *p*-value of < 0.05. Our findings are in agreement with the suggest that suggest biofilms to be associated with increased resistance to antibiotics [23–25].

Previous studies have also indicated that biofilm-forming bacteria tend to exhibit higher resistance than planktonic cells due to the tough polymeric matrix that impends antibiotic penetration [26].

In this study, biofilm-forming organisms showed marked resistance to most commonly used antibiotics such as Ciprofloxacin, Ceftriaxone, and Gentamycin. This was also similar in a study by *Neupane* et al [12]. These findings underscore the need to regulate the use of antimicrobials and institutionalization of antimicrobial stewardship programs in hospitals to limit the spread of resistant microorganisms [27]. In Uganda there is no regulation of use of antibiotics with patients having access to over the counter prescriptions, this has been one of the major drivers of antibiotic resistance in the communities [28].

In the present study the prevalence of *Fim, Pap, Sfa, Afa, Hly and Cnf genes* in UPEC were determined and the results showed that among biofilm producers, *Fim* was the most prevalent Urovirulence gene followed by *Pap*, *Sfa*, *Afa* and *Cnf*. This was similar to other studies [29, 30]. In our study biofilm production was not associated with any of the virulence genes or resistance to any particular antibiotic. This was however different from other studies. A study by *Manuela* et al. reported that biofilm production was significantly associated with fluoroquinolone resistance [30]. In another similar study by *Zamani* et al it was found out that biofilm-producing UPEC were significantly associated with *Fim* gene [31].

## Conclusions

This study demonstrated a high tendency among the UPEC isolates to form a biofilm. The biofilm-forming organisms were more MDR, produced more virulence genes compared to the non-biofilm forming organisms. Therefore, knowledge of biofilm formation, virulence factors and their antibiotic susceptibility pattern is key to guide the management of patients with biofilm associated infections.

## Supplementary information

**Additional file 1.**

## Data Availability

All data generated or analyzed during this study are included in this published article [and its supplementary information files]. DOI; 10.6084/m9.figshare.11321936 Fig share.

## Abbreviations

CLSI: Clinical and laboratory standards institute
DNA: Deoxyribose nucleic acid
MDR: Multi drug resistance
PCR: Polymerase chain reaction
SOPs: Standard Operating procedures
UPEC: Uropathogenic *E. coli*
